# Muriel Spark’s ‘informed air’: the auditory imagination and the voices of fiction

**DOI:** 10.1080/0950236X.2018.1533171

**Published:** 2018-11-21

**Authors:** Patricia Waugh

**Affiliations:** Department of English Studies, University of Durham, Durham, UK

**Keywords:** Voices, auditory imagination, phenomenology and ontology, Apophatic and Kataphatic, group psychology, ethics of fiction

## Abstract

‘Events occur in my mind’, Spark has written, ‘and I record them’. What does it mean to hear something that isn’t there? Hearing inner speech or sounds, not as silent thoughts but as quasi-perceptual events in the world, confounds settled distinctions between perception, memory and imagination that structure our feeling of the real. This essay shows how her capacity for complex ‘listening in’ becomes the mainspring of her brilliance as an experimental writer, cultural observer and fictional ethicist. Eclectic in her sources – including biblical, classical, Romantic, Christian and Jewish mystic and monastic traditions of meditation – Spark reworks the concept of ‘the auditory imagination’ to produce one of the most sustained and innovative self-reflexive performances in modern fiction of the human mind’s capacity to bring to presence and realise other worlds, multiple ontologies. In her writing, fictional vocalisation is the phenomenological and expressive vehicle of an embodied ontology of plural uniqueness that opposes metaphysical reduction to a universal One that invisibly eradicates difference.

In T. S. Eliot’s idea of the auditory imagination, the gift of a ‘feeling for syllable and rhythm, penetrating far below the conscious lives of thought and feeling’ is able to fuse ‘the old and obliterated and the trite, the current, and the new and surprising, the most ancient and the most civilized mentality’.^1^ Some twenty years later, Muriel Spark set about its refashioning in her own inimitable style. In 1953, after publishing a review of Eliot’s *The Confidential Clerk,* she ‘embarked’ on a book-length study for the Catholic publisher Ward and Sheed (it would never, in fact, see the light of day).^2^ She was preparing to enter the Catholic faith. Coinciding with this life-changing decision was another life – as well as writerly – change of tack: the transition from Romantic critic and poet, to become one of the century’s most innovative experimenters with voice in fiction. These are the years when Spark found her own voice or, more accurately, her voices. But this moment of radical transition was fraught: her auditory imagination was to be refashioned in an encounter with Eliot that carried existential ramifications resonating – a far cry – beyond merely aesthetic conceptualisation.

Spark’s writerly sense of and preoccupation with voice intensified as she made the transition from poetry to fiction, but other events in her life played a crucial role. Sometime between 1953 and 1954, she came to believe that Eliot was ‘communicating’ secretly with her, sending her covert messages in his writing which, in her autobiography, she refers to as ‘ my hallucinations’.^3^ The decision to transmute her experience into Caroline Rose’s more direct experience of ‘hearing voices’ in *The Comforters* was, she insisted, purely pragmatic, easier to do as voices.^4^ In objectifying the feeling of heightened significance in Eliot’s words through a consciously controlled process of personification (the typing ghost), Spark began to reflect on the power of the human imagination to create feelings of presence that might challenge or vie with those of external powers of perception. (Voice hearers too often communicate their experiences as ‘felt presences’: ‘I heard them without hearing them’.^5^) Eliot’s words had taken on for her a presence of Kabbalistic intensity, blurring distinctions between seeing with the external eye and hearing with the mind’s ear. Reflecting on the experience shortly after as she resolved to write a novel (her first) about her hallucinations, she became convinced that the novel ‘as an art form was essentially a variation of a poem … an extension of poetry’.^6^ Her sense of this was confirmed later when she stumbled on a piece of ‘dialogue-criticism’ between the American critics Mark van Doren and Allen Tate: prose narrative, Tate suggests here, becomes poetic when ‘it deals with action conveyed through fictions of the imagination’.^7^

For Spark this ‘magic piece of dialogue’ was confirmatory of her own sense that ‘all my hallucinatory experiences, looking back on them, seemed to integrate with this idea’: *The Comforters* would explore how a novel might be an extension of a poem and the entanglement of this process with her conversion to Catholicism and the hallucinatory experiences of her breakdown.^8^ Arriving simultaneously, such radically disorienting experiences seem to have driven a desire to explore, question, and open up new thinking around the possibilities, connections between, and understanding, of the mysterious human propensity for imaginary presencing: as the source of art, as the religious experience of encountering God, and as the terrifying dark night of the soul, the madness of the self abandoned and evacuated. For Spark, as she reinvented herself as a novelist, it meant too a reflection on the peculiarly auditory nature of those experiences and on the relationship between hearing voices and ‘voice’ in fiction and poetry. In an interview some years later, she would insist that ‘all poets hear inner voices … rather than seeing visualized scenes’: but what does it mean when the poet hears inner voices as outer communications and then transitions into a *poet-novelist*?^9^

Spark’s terror at this time – that she would lose her mind and be unable to communicate her life-altering experience – is conveyed in the title of the novel, which alludes to the sufferings of Job and the ineptitude of his literal-minded and ineffectual comforters in their imaginative failure to empathise with his distress or the nature of his dialogue with God. Most likely, it also references Yeats’ 1913 poem ‘The Realists’, whose Job figure is the modern poet as mythopoeic diviner, marginalised and misunderstood in a culture of fact-mongering and reductionist naturalism.^10^ Spark invents Caroline Rose as a means to explore disturbing experiences of possession by voices or thoughts not her own, appearing to ‘speak’ with an alien volition that deprives the self of agency. If Eliot appeared to be directing her thoughts at this moment, some version of Tausk’s famous ‘influencing machine’ would continue to reappear in her later novels (most explicitly again in *A Far Cry*, the novel that returns to this period of her life, with its radionics ‘box’, its organisers and operators, that are instrumental in the suicide of Wanda).^11^ Caroline Rose’s is a ‘mind working under the pressure of someone else’s necessity’,^12^ but her voices speak in the unspeakable or aoristic voice of fiction: the novel plays out continuously looping self-reference to its own emergent space of composition.

In ‘My Conversion’, published a few years later, Spark describes her mind at this time ‘teeming with disorder’, but shortly after came the recognition that ‘it was like getting a new gift’.^13^ This is not Edmund Wilson’s romanticised and theodicial ‘wound and the bow’.^14^ The ‘gift’ of the breakdown is more complex than that and more personal. Certainly it was bound up with a sense of Grace and with her conversion, the feeling that her imaginative capacity was a gift from God. But always she had been aware, in encounters with music, art and poetry, of a ‘definite “something beyond myself”. This sensation especially took hold of me when I was writing; I was convinced that sometimes I had access to knowledge that I couldn’t possibly have gained through normal channels’.^15^ In writing about her breakdown in 1961, it might appear that she had come to view her conversion as ‘the gift’ that delivered her because it resolved her sense of existential crisis and offered a new beginning as an artist. It might appear as though she had come to see the conversion also delivering her into a new sense of unitariness as a self, a transmuting and harmonising of the teeming inner and outer voices into one voice:

Nobody can deny that I speak with my own voice as a writer now, whereas before my conversion I couldn’t do it because I was never sure what I was … I was talking and writing with other people’s voices all the time.^16^

What I will argue here, however, is that although, after her conversion, Catholicism became what she referred to as a ‘a norm’ organising her life, preemptive harmonisation is not what she had in mind. Departing from the norm was the ‘nevertheless’ principle in operation, and the discovery of her own ‘voice’ might be deemed to be the discovery of an even greater aesthetic and ethical requirement to listen and to discern, not to evacuate or repress the voices of others heard in one’s own.^17^ Indeed, the conversion and the breakdown together provided the means of recognising others’ voices as parts of and yet singularly other than her own: they provided a very direct experience of herself as radically disaggregated and yet emphatically singular, self as a many in one. Together they gave her the opportunity to find her own ‘voice’ as a novelist and a self as a plurality of ‘voices’ – and to exploit the resources of that most dialogic of genres in her remodelling of it – as the realisation of a principle of radical alterity at the heart of the self. In her writing thereafter, the self is conceived as a steady state of disequilibrium, an ever emergent and potential entity, never context-independent but always context sensitive, inherently multiple and dialogic, most public and social when it is most private and intimate. The ‘gift’ is the recognition of her capacity to tune in to the voices of others, heard externally but also in the inner and mostly subterranean dialogue of self with itself and to recognise therefore that her future as a writer was bound to the novel genre.

It was a gift whose powerful ethical import was quickly recognised and would shape her sense of the ethical possibilities of the novel and the social responsibilities of the novelist. The eschewing of an art of harmonious and empathetic identification – effectively the incorporation of the other into the self for an art of satire, ridicule and disturbance – called for in her essay of 1971 on the ‘desegregation of art’, seems to have fully materialised during her attendance for *The Observer* newspaper at the Eichmann Trial in 1961.^18^ Though she returned in a state of shut down horror, unable to write the promised report, verbatim transcripts of the trial appeared in *The Mandelbaum Gate* (1965). As the novel makes clear, like Arendt’s *Eichmann in Jerusalem* (1963), Spark’s sense of horror was provoked by the incantatory monologism of Eichmann’s voice, a ‘computing machine’. ‘What was he talking about? The effect was the same in any language … and the actual discourse was a dead mechanical tick’: even without semantic grasp of what is being said, the sound of the voice condemns him,^19^ conveys his inability to think, because thinking, if it is to be reflective and ethical, is to think in and hear the voice of the other in one’s own. The shattering experience of listening in through the electronic earpiece to the voice of Eichmann: the man in his glass cage, the voice, interminable, self-absorbed, as monstrous as it was monologic, resonated, to prompt, in *The Mandelbaum Gate* (1965), a recognition of how much pluralisation of voice even as it performs a kind of precarious disequilibrium with conventional ideas of unity, harmony and oneness of selfhood, must become the fundamental ethical vehicle of her fiction.^20^ The banality of evil is a bureaucratic or mimetic monologism that cannot hear the voice of the other. It is thoughtlessness, thought as devocalised solipsism.

*The Mandelbaum Gate* is the novel where, after Caroline Rose in *The Comforters*, Spark most directly poses the question, ‘Who am I?’ through her character Barbara Vaughan, who replies to herself, ‘I am who I am’: all three ponder their condition of ‘Gentile Jew’ as well as Catholic convert.^21^ To grasp the full complexity of Spark’s auditory imagination requires keeping in mind that although her conversion, breakdown and transition to fiction coalesce in the events of 1954–1955, the idea of conversion as a sudden and irrevocable ‘event’ that entirely casts off a former self (in some versions of the Augustinian) can be misleading. The idea too that Spark found her ‘voice’ in the conversion to Catholicism as the only catalyst launching her novelistic career, is a convenient (and conventional) simplification that fails to do justice to the joyous and celebratory but also paradoxical poetic-novelist performances of her art. The auditory tendencies of her imagination had already, in 1954, had a quarter century distillation. In *Curriculum Vitae*, she describes herself, as a child, as ‘an avid listener’, ‘I liked to listen’.^22^ From her earliest years she became acutely aware and sensitive to (the sensitivity of the child to perceived exclusions from the group) of aural difference, of idiolect and sociolect, through the markedly different and despised English accents of her mother and the (Russian) Jewish heritage of her Scottish father. In the 1930s, the variety of Edinburgh voices of her school years fed her delight, in particular, the ‘dazzling’ non-sequiturs of the charismatic teacher, Miss Kay, the prototype of Jean Brodie. In the last years of the war, her work for the Political Warfare Executive, whose operations consisted of listening in, to radio and telephone, and of mimicking voice, in broadcast and broadsheet, taught her to listen in attentively to the ‘scrambler’ – ‘to listen “through” the jangle' – so that, years later, she recognized someone as a voice from that era, without ever previously having seen the face.^23^ Spark’s wider intellectual and artistic interests find congruence with these patterns of experience: the early interest in and writing on voice in Romantic poetry; her own self-proclaimed dialogical identity of ‘Gentile Jewess’ and abiding interest in the voices of the Old Testament and in Christian and Jewish spirituality;^24^ her continued fascination with listening in and hearing voices, with spies, imposters, mimics, aiders and abetters, all those who operationalise a mode of imaginative presencing that is not simply the provenance of the psychotic, but shared by the artist, the mystic, the newly bereft and the converted.

Although Spark’s is not the only ‘auditory’ poetic imagination in the twentieth century – similar claims might be made for Kafka, Joyce, Bowen, Proust, Joyce, Woolf, Richardson, Conrad, Auden, Eliot himself and many more since – she is perhaps the most persistent in her claim to be characterised thus. Attention as ‘listening in’, throughout her *oeuvre*, might take the form of inward self-meditation, aural mind-wandering, absorbed listening, or the intent eavesdropping that follows the sudden awareness of salient vocalisation in the midst of noise that psychologists refer to – no Eliot pun intended – as the ‘cocktail party’ effect . Spark’s fiction plays, comically and mischievously, with mishearing, overhearing, aural misconstrual, in order to expose the interpellating pressures of sociolectic constraint. But ‘listening in’ is also Spark’s figure for memory in its entanglement with imagination. In *Loitering with Intent*, the writer-protagonist, Fleur Talbot, insists that: ‘If I recall certain encounters of the past at all … back come flooding the aural images first and the visual second.’^25^ Echoing Wordsworth’s delight in how ‘my own voice cheered me’,^26^ Fleur too sees ‘no reason to keep silent about my enjoyment of the sound of my own voice in the work’.^27^ Spark’s fictional turn in the 1950s prompted a reconsideration of imagination as a mode of inner sensory ‘presencing’ with a distinctive focus on the mind’s ear.^28^ So *The Girls of Slender Mean*s opens with a familiar scene of Romantic visualisation: the (wartime) fragment, the ruined building, but this makes, the narrator concedes, ‘unusual demands on the mind’s eye’.^29^ Shells of bombed tenement buildings, with their lavatory chains surreally dangling over nothing from fourth and fifth floor ceilings are self-consciously presented as a scene in the theatre of the mind: ‘exposed, as on a stage’, the effect oddly one of intactness and distance. Uncannily vivid yet removed, they feel no longer real.^30^ Then the telephone intrudes. The voice of Jane Wright breaks in, now a fashionable magazine journalist, breathlessly eager to impart the newsworthy and the sensational, to gather into the present the long gone community of the past: *discours* overwhelms *histoire* as the present shifts to that of narration rather than narrated, bringing with it the first hint of diegetic violation, of metaleptic complication, with her announcement, ‘I’ve got something to tell you. Do you remember … ’. In Dorothy Markham’s ‘Darling, where have you been?’, is heard the fading phatic communion of her set.^31^ But it is voice more than visualisation that brings the past into presence as the auratic architecture of the long razed house is brought to life again, the building a ‘euphony of birds’ chirping into life, the resurrection of the dead.^32^

Virginia Woolf too had described the creative process as one that begins with an intent and absorbed listening in: ‘Instead of remembering here a scene and there a sound, I shall fit a plug into the wall; and listen in to the past.’^33^ But Spark’s favourite metaphor is of ‘tuning in’: ‘I was tuning into voices without really hearing them as one does when moving from programme to programme on a wireless set’, writes Fleur in *Loitering with Intent* (whose very title, explored in the opening pages, is an image of the writer as a kind of spy, one who loiters, listening out and in, for hidden purposes of her own).^34^ Inverting the ‘mind mindedness’ of maternal care, the empathetic ability of the mother to intuit her baby’s needs, the narrator of an early story, ‘The First Year of My Life’, describes post-natal telepathic powers that enable her to ‘tune in’ to world events as if listening to a radio in her head and to the thoughts and feelings of her caretakers. The story effects a playful *reductio ad absurdum* of the claims of the Romantic imagination to that anamnesic power that makes the child father to the man.^35^ In the ‘author’s note’ to *Voices at Play* (1961), too, Spark again describes how ‘I turned my mind into a wireless set and let the characters play on my ear’.^36^ Many of them, like Sandy Stranger disengaging from the whine of Miss Brodie’s voice as she listens in to the more compelling voices of her own imaginary characters, hear with double ears: the outer ear of perception and the inner ear of imagination.^37^ In her critical study of Mary Shelley, Spark asserts: ‘I know now that I have a “writing ear”, that is the act of imaginatively getting under the skin of a character that produces the individual character’s diction.’^38^ Derek Stanford was the first to note her ‘artist’s cultivation of the ear’ but also to suggest that the secondariness of her visual imagination sometimes meant that character suffers, ‘the coincidence of voice and person is lost’.^39^ Stanford fails to recognise how that technically achieved loss is actually the mainspring of Spark’s experimental aesthetic.

Penelope Jardine has suggested that ‘something in the air’ was ‘a phrase she used to explain how things come to us. That certain, or almost certain feeling of something’s existence that most artists know when creating. It must be there. How else did they think it’.^40^ Jardine intuits the uncanny nature of the experience of sensory perceptions, of thinking and feeling as presence, when they occur in the absence of external stimulus of the sensory organs. Hearing inner speech or sounds, not as silent thoughts but as quasi-perceptual events in the world, confounds settled distinctions between perception, memory and imagination that structure our feeling of the real. ‘Events occur in my mind’, Spark writes, ‘and I record them’.^41^ But what does it mean to hear something that isn’t there; and what does ‘there’ mean in this instance? In Spark’s third novel, *Memento Mori*, the acousmatic authority of the anonymous phone caller derives from its functions as a Maxwellian demon, but one who unsorts and unseparates – the imaginary and the real, the living and the dead – calling up a fragile community of souls in the call to mindfulness of ‘Remember you must die’.^42^Spark was reading Proust shortly before the novel’s composition and no doubt had been impressed with the unsettling incident where Marcel, listening in to the disembodied telephone voice of his grandmother for the first time, hears, not the comforting and homely voice that has been the guarantor of his childhood world, but instead, something alien, deeply cracked and moribund.^43^ He is overwhelmed by a future anterior sense of the doubly absent, a living already part of the dead: ‘a real presence’ Marcel thinks, but also ‘a premonition of an eternal separation … I have felt the anxiety that was one day to wring my heart when a voice would thus return (alone and attached no longer to a body which I was never to see again)’.^44^

Spark refigures the Proustian voice in the concentration camp survivor Cathy in *A Far Cry from Kensington*, cackling through the office din, ‘with her terrible voice’.^45^ But here, the voice is the register of that mode of absence from oneself that is the condition of the survivor, the savagely traumatised, and abjected. The faraway cry of Wanda, the Polish refugee from thirty years ago, returns in memory, too close for comfort: ‘I had in my mind’s ears that cry of Wanda’s … That cry, that cry.’^46^ The screaming of the abject is heard in humans who emit barks, in laughter, maniacal in *The Driver’s Seat* but in *Not to Disturb* antic, indifferent, ‘sunlight laughing on the walls’ ushering in a new world of the mediated and sensational, of technopastoral opportunism.^47^ Throughout Spark, voice – embodied or disembodied – is firstly heard not as the iteration or reiteration of semantic meaning, but as the physicality of sound upon the ear. In her worlds, primary listening begins with an attention to what precedes speech, antecedent to the semantic. In this distributed network of agency, objects speak too; intentionality is never to be located in the coincidence of bodies and voices: time, space, technology, things, always intervene. Spark defies realist expectations, as she defies Aristotle’s description of *logos* as *phone semantike*, his insistence that man is a creature who has *logos* because only he speaks in language.^48^ Typewriters speak in *The Comforters*, or buildings, ‘bleating to high heaven’;^49^ in *The Abbess of Crewe*, walls have ears and so do trees; in *The Girls of Slender Means*, radios ‘speak’ to listeners rather than transmit sounds; in *The Hothouse by the East River*, the glittering city comes to Elsa ‘agitating all around her ears’, like Augustine’s coming to Carthage, city of unholy loves, that ‘bubbled around me in my ears’.^50^ In *A Far Cry*, ‘the household’ is ‘garrulous with tacit deliverance’,^51^ but voices rise up with singular force bringing a glimpse of other worlds. The vehicle of the simile that introduces the voice of the treacherous Sir Alec Tooley is inflected with a tenor that gives rise to urban pastoral, the hint of other and better worlds, his voice, ‘like a wisp of smoke wafting from some burning of leaves hidden by a clump of lavender’.^52^

Throughout her writing, voices as conduits of absent presence are the instruments of ontological leakage and liberation, of what Latour names the ‘factish’, awakening the mind to the possibility of other worlds. Voices enable ‘a wisdom of passage’ from the either/or options of the modern repertoire to the thought-worlds of the pre-modern, challenging narrow conceptions of the rational and the real.^53^ In Latour, the ‘factish’ unsorts the assumption that otherworldly belief is necessarily fetishistic or simply magical thinking by revealing the origins of even the most scientific facts in collective practices that have disguised their own artefactual operations.^54^ In Spark, the factish operates to subvert the hegemonic through creative and often comic scrambling and mishearing of language: ‘Garble is what we need, now, Sisters … Mythology is nothing more than history garbled’; ideology is delivered unhinged.^55^ The human capacity for what Eugen Bleuler, the phenomenological psychiatrist, called ‘double book-keeping’, for living in more than one world at once, rests on the creative misprisions of the ear.^56^

## Presence, absence and the pluralisation of voice: phenomenological metafiction in Spark’s early writings

In Eliot’s version of the auditory imagination, the idea of vocal inwardness as the location of deepest selfhood is still Romantically inclined. It is a version of Plato’s soul in silent communion with itself. The image recurs, playfully, in Woolf’s short story, ‘An Unwritten Novel’, as the narrator asks: ‘When the self speaks to itself who is speaking?’. Playing with the Freudian theory of repression, she goes on to suggest the interlocutor might be the ‘entombed soul’, the one ‘that took the veil and left the world’.^57^ Woolf, ironically, Eliot more nostalgically, divine a yearning for the idea of a more unified selfhood, deeper than grammar or reason. For Spark, however, the auditory imagination reveals not a deeper at-oneness, but the inherent dialogicality of outer and inner speech, voices, emphatically pluralised, oriented and responsive, brought into being by visible and invisible interlocutors. ‘Thought’, she writes, ‘is the main ingredient of a creative work, and thought goes with intention of some sort’.^58^ In the dialogic auditory imagination of the genre of the novel, and especially in the highly experimental dialogism of this particular novelist, however, intention, like agency, is multiply distributed.

Caroline’s experience is of a voice ‘like one person speaking in several tones at once. They speak in the past tense. They mock me’.^59^ But careful listening reveals a plurality of voices: ‘It was impossible to disconnect the separate voices, because they came in complete concert; only by the varying timbres could the chorus be distinguished from one voice.’^60^ Consideration of poetic hearing of others' voices in evident throughout earlier Spark’s earlier poetry and criticism, appearing explicitly in her book on John Masefield, for example, who is described ‘trying to speak through a hum of other voices which, none the less, he felt the need to listen to’.^61^ In Spark, vocalisation is the phenomenological and expressive vehicle of an embodied ontology of plural uniqueness that opposes metaphysical reduction to a universal One that invisibly eradicates difference. Discovering this led her to reject realism as dogmatic, and Romanticism as an over-preoccupation with the One. For a realist metaphysics, grounded in empiricism, with objectivity its avowed ideal and a correspondence model of truth its expression, to hear a voice in the absence of an external interlocutor or verifiable source, or to believe as true what no external perceptual mechanism can locate, is simply to be in error.^62^ Two options only are on offer: truth which is a transparent reflection of this real, or error when that real is skewed. To take the imagination for the real is the most dangerous error of all. The modality of perception is the touchstone of this real; its primary sensory organ is the eye.^63^ In this ontological framing, there is no room for ambiguity, no in-between states. The human capacity for inner presencing, for the things of imagination to take on the qualities of the perceptual, is viewed as the predilection of the untrained mind, prone to delusory aberration, at risk of derangement and psychosis. But Spark’s work is fascinated with how that inner sensory world may override the external senses, dislocating the here and there, then and there. If memory too as a conduit for imagination overrides external perception, one becomes adrift in time, neither now or then. This doubling of time and space – the feeling of ‘where am I?’ – is also an effect of the human capacity for absorption in fictional worlds.

In metaphysical and scientific traditions, modalities of intentionality are hierarchised: perception, memory, imagination. So too are the senses. The eye is the realist and metaphysical sense par excellence, looking on evidence laid before it. Sound is ambiguously located, blurring inner and outer senses, always in movement, conveying space as temporality, fading out, surrounding or coming towards. In listening, the ear is a transduction device that transforms the vibrations of air as they hit the tympanum: the ear and the imagination are organs and modalities that disturb embodied proprioception, the sense of the body and other bodies as here or there, now or then, inside or outside; with this comes the destabilisation of metaphysical and existential certainties. In sound, the unseen speaks; a beyond reverberates past the horizon of vision.^64^ Where the metaphysical eye seeks causes, the phenomenological ear proposes an open and purely descriptive ontology: ‘I don’t go in for motives’ Fleur says, ‘I never have.’^65^ In Spark’s fiction, the realist as copier, the rhapsode as parroter, and the Romantic as one-eyed visionary, are those who represent a thanatic impulse of control and containment, a solipsistic inability to listen and discern, to respond to the cry of the other. Such characters are all ‘pisseurs de copie’,^66^ sloppy mimeticists and fraudsters such as Hector Bartlett in *A Far Cry* (who is instrumental in Wanda’s suicide) or like Lise, in thrall to a darkly one-eyed Romantic model of the imagination where the scene of composition is already unfurled in the moment of inspirational conception; they attempt to preside over worlds where the imagination seeks to bring the real into accord with its prearrangements. They live perpetually and metafictionally, like the canny sex-and-death-driven servants of *Not to Disturb*, in the mode of the future anterior.

The auditory and the vocal in her novels serve as the phenomenological foundation of an anti-metaphysical aesthetic that promotes ontological pluralism and an ethics of radical alterity, facilitated not through vision but through cultivation of the discipline of attentive listening. Recent critics^67^ have begun to note this anti-metaphysical import of her vocal imagination but early admirers, even the astute Frank Kermode, failed to notice the ontological and ethical ramifications of this pluralisation of voice. Kermode describes her work as ‘always a monologue’; her characters ‘all speak in some version of her voice’.^68^ But Spark was responding to a variety of assertions of and assaults on the vocality of presence in the middle years of the century. One was McLuhan and Ong’s campaign for a return to the spoken voice in an era of ‘second orality’ bringing back the ‘word as presence’.^69^ The new sonar technologies of information distribution and transmission might now facilitate this newest revolution of the word. Spark was less sanguine. She knew from her time in the PWE that Shannon and Turing had developed the vocoder or ‘scrambler’ (featured in *The Hothouse by the East River*) that was first tested using Churchill’s voice.^70^ The reference is picked up in *The Girls of Slender Means* where Nicholas Farrington’s enjoyment of Joanna’s rhapsodic performance of ‘the mountains look on Marathon’ from Byron’s ‘The Isles of Greece’ is ironically ‘interfered’ with by Churchill’s reactionary and disastrously misjudged ‘Gestapo’ broadcast of 6 June 1945. This had revealed his complete failure to hear the new democratic mood at the end of the war. Electronic mediation, interference, hissing on the line and scrambling, are ways in which Spark resists the McLuhanite optimism of a ‘second orality’: rather than a medium for a new communitarian presence, the nuance of the voice in this electronic world becomes even harder to discern. Sefton Delmer too describes the operations centre of the PWE in the last years of the war as a ‘Tower of Babel’.^71^

Within the literary academy, however, another revolution was afoot whose rationale was an assault on voice as presence: the Derridean critique of orality as underpinning a dangerous metaphysics of presence. As Nancy/ formerly Mrs Hawkins observes, ‘we tend to notice what we want to’: for the literary critic presence and orality would soon fall out of earshot; writing was making its comeback.^72^ Derrida’s critique of voice as the foundational concept of metaphysics reflects his fear that inner voices create an illusion of the integrity of interior space as a presence to itself guaranteeing the autonomy of the sovereign self: here we are now, here we are deep within. In the post-structuralist critique, not speech, but ‘writing is the destruction of every voice, of every point of origin’.^73^ The self-consciously writerly text must devocalise and therefore disrupt the tradition of metaphysical presence underpinning Greek, Christian, Natural Law, Romantic and later existential and phenomenological accounts of truth. For Spark, however, voice is a disrupter of naïve self-presence. Plato’s association of orality with presence, in his own critique of writing, required that speech be heard as semantic and warranted, so as to separate the voice of reason from the rhapsodic, the emotionally contagious performance of poetry.^74^ Plato’s is an orality already stripped of vocality. Derrida’s critique of speech makes too little of Plato’s relegation of the physical voice, the ‘sweet singing art’ that the Muse bestows upon the blind Homer in Book 8 of the *Odyssey*. For the philosopher must tremble if the poet, alone of all men, has the gift of listening to and understanding the oral testimony of the Muses, daughters of Mnemosyne, uniquely able to hear and reshape her sounds into patterns of meaning discernible to the attuned layperson’s ear.^75^

*The Comforters* has largely been read in this Derridean tradition of absence and writerly reflexivity as a prime example of early metafiction: ‘a novel about characters in a novel’ in Caroline Rose’s own words.^76^ Yet the act of listening is foregrounded from the very beginning, as Laurence Manders, ‘sleuth-like’, strains his ears to the floorboards to spy on the activities of his grandmother, Louisa Jepp, as she moves about her kitchen.^77^ His listening in behaviour also configures a metafictional doubling: Spark’s figure for the activity of the writer in creating and inhabiting a complex space of composition that emerges dialectically as process. Laurence, however, whose job is ‘on the wireless’, his ‘intelligence’ doubtful in his grandmother’s eyes, approaches the auditory merely as anticipatory clues to a reality to be validated through visual confirmation.^78^ Over-precipitately, he translates what he hears into what he thinks he sees: throughout Spark’s work, the tendency to confirmation bias through mishearing is summed up in the phrase, ‘people notice what they want to’. One of the literal-minded, Laurence is the first and most intimate of Caroline’s comforters, a realist trapped in a metafictional novel whose ontological openness perpetually defies his grasp.

The novel abounds with metafictional devices, particularly those diegetic violations that create disturbing effects of metaleptic instability: what world is this taking place in; what world are we, as readers, and they, as characters, experiencing? Louisa Jepp, one moment securely rooted in a second order diegetic frame of the storyworld (for the reader, temporarily, a first order world), in the next has broken into the ‘real’ of the assumed first order historical present of narration, so reminding the reader of her other ‘reality’ as a verbal construct. She is ‘still alive’ but ‘here in her home in Sussex, now in the present tense’.^79^ The space of composition is continuously introduced through self-referring similes that keep the presence of the activity of writing at the edges of the reader’s awareness: ‘a whole day unplanned … like a blank sheet of paper to be filled in according to inspiration’.^80^ Even the many-in-one nature of the voices is a reflexive performance of free indirect discourse, the unique ‘voice’ of writing foregrounded as a second order assigning of speech to a figure concretised at a first order of narrative discourse. The peculiar oneness and plurality of the free indirect voice in fiction in its capacity to entangle narrators and characters normally positioned at different diegetic and therefore reality-effect levels, is here metafictionally flaunted to open up ontological possibility. The typing ghost voice figures an allegory of composition that points to how the gradual liberation and emergence of voices into characters in the fiction-producing process inevitably alienates the writer who must also become a reader of herself: ‘outside it, and at the same time consummately inside it’.^81^ There is no initiatory, unitary intentionality, above, below, outside, determining the text and pre-emptively figuring its final shape.

So far so postmodern. What happens though if, instead of foregrounding the metafictional elements as simply a novel about writing a novel, *The Comforters* is approached as an investigation into the complex and many ways in which the presence of the real is felt in our lives as well as in our fictions? What if the text is approached less as a Derridean play of textuality, more as a phenomenological metafiction that builds on Spark’s preoccupation with hearing and listening as modes of existential presencing? Even the flaunting of character – and by the end, the text refers to ‘the character called Laurence Manders’ – as text and ethos overturns the usual assumption of the priority of persons, to suggest that treating persons as characters is not necessarily an act of colonisation, limiting and condemning them to a ‘phony plot’, but potentially a liberating challenge to assumptions concerning the metaphysical fixity of the self. The metafictional devices are used to convey the kind of phenomenological perplexity undergone in the experience of hearing distressing voices, but in order to prompt further reflection on the kinds of inner presencing associated with different modalities of intentionality, including those involved in creating and entering fictional and other kinds of worlds. As Caroline feels increasingly alienated from the Catholics of St Philomena’s and the *demi-monde* world of louche Bohemians of her past, she begins to experience an inwardly focused distress that turns her thoughts on themselves so that language – as in Spark’s Kabbalistic experience with Eliot’s words – begins to take on a strangely concrete presence and personifying elaboration. Even before the voices appear, Caroline increasingly objectifies her inner speech, talking to herself, then experiencing language as material, another’s thoughts and voice inserted into her own: ‘as if the person was wanting to pounce on some insignificant thought or action, in order to make it signify in a strange distorted way’.^82^ Spark explores how the absorptive capacities of mind regulate or disregulate its relations with the world through shifting intensities of attention.

The novel’s retelling of Job’s suffering reveals the inadequacy of excluded middle thinking that proposes an either-or model of truth: one bound to epistemology, realism and objectivity, the other viewing truth as enigma. The various supernatural, black magic, quasi-scientific, moral, even conventionally religious explanations for Caroline’s suffering are revealed as failures of the empathetically disciplined imagination and serve to increase her distress: ‘this is a very remote world I’m in now’, she tells Laurence.^83^ They are bound to a regime of truth that sets the empirical fact against the supernatural fetish as domains of non-overlapping magisteria. In the world of the comforters there is no ‘factish’. Laurence, who ‘terrorised the household with his literal truths’ and whose relation to language is obsessively to mimic and expose clichés, is the first of Spark’s many reporters, rhapsodes and parroters, incapable of imaginative liberation from a positivist relation to words.^84^ But the priest, Father Jerome too, can only mimic God’s words to Moses, ‘I am what I am’, as he advises acceptance of mystery, ‘You are what you are’.^85^ Caroline, believing that she can change her relations in this world, rebels with her own subversive mimicry. ‘I *am* in retreat’, she thinks punningly at St Philomena’s, reflecting on the banality of the obligatory phatic communion that consolidates one’s attachment to the group. In that skittish utterance alone, she liberates herself from thralldom to it.^86^

The diegetic play with worlds relates phenomenological disturbance to questions of ontological possibility and existential threat, but Spark further liberates the resources of the novel to begin to explore more ethical, social and political issues around identity, exclusion and belonging. The invention of the arrestingly non-realist character, Mrs Hogg – ‘an implausible character’,^87^ ‘not a real life character … only a gargoyle’ – is the figural crux for examining such relations.^88^ Morally repellent (to Caroline) and intrusive, her presence prompts Caroline’s urge to brush her teeth; Caroline watches Hogg chewing rhythmically to the sounds of the liturgy and experiences her as a bolus of food stuck in her gullet. Never intended to be a realistic character, ‘pathetic and lumpy as a public response’,^89^with ‘no private life whatsoever’, Hogg yet claims to be a voice hearer, attributing all of her life decisions to a special capacity to hear the guiding voice of Our Lady. She is a projection of Caroline’s profoundly negative but largely disavowed sense of alienation from the group, her revulsion from and fear of absorption into the lives of flesh and blood Catholics: ‘Catholics and Jews; the Chosen, infatuated with a tragic image of themselves’, she thinks at St Philomena’s,^90^ while her experience of the Brompton oratory is of a ‘terrifying collective’.^91^ Paradoxically Caroline’s singularity is only and terrifyingly to be preserved through the manifestation of the typing ghost with its many voices in one.

## *Ruah* and the vocal imagination

That the intellectual framework of Catholicism shaped Spark’s sense of fictional form is well known; Spark’s conception of voice as a plural singularity, however, allowed her to accommodate and transfigure the many sources of the auditory in her life and, like Eliot’s, to accommodate the trite, the current and the ancient.^92^ Part of this process was her interest in spiritual exercises that make present the divine: the Apophatic, as in the ‘cloud of unknowing’, invoked in *The Mandelbaum Gate*, *The Hothouse by the East River,* or the Kataphatic presencing of monastic traditions of prayer and meditation.^93^ Her familiarity with the spiritual exercises of Ignatius, especially the idea of ‘the composition of place’ in the meditations for the first day – building and placing yourself in the inner scene in the presence of God – would have familiarised her with the process of achieving a sense of presence through inner sensory focus or attentiveness, recovering the imagination that Ignatius associates with the child, with its capacity for inner hearing, seeing, smell, touch and taste.^94^ The Kataphatic tradition emphasises hyperattentive inner focusing that is likened to the composition of a mental architecture, a house fit for characters. But in Jewish traditions of spirituality, especially, it is the power and physicality of the voice that is the prime vehicle for this sense of presence. In the mid-century, a revival of this tradition was ongoing in the writing of messianic Marxists such as Bloch and Benjamin and radical phenomenologists and political thinkers such as Levinas, Weil and Arendt. All were searching for new models of ethical and political imagination. ‘The scriptures were especially important to the half-Jew turned Catholic’ reflects Barbara Vaughan in *The Mandelbaum Gate*.^95^

They were important for Spark too; for voice (*Ruah*) is central in Jewish spirituality. That *Ruah* as the physical breath of Elohim precedes speech means that in the beginning is not a devocalised logos but instead the vital spirit of God sounding over the face of the waters. Similarly, *Qol*, the sonorous self-revelation between God and the world that becomes speech in the mouth of the prophets, begins with the physicality of the singular voice.^96^ Spark’s interest in this Hebrew understanding of voice is evident in her refashioning of the auditory imagination in the *Observer* Christmas prize-winning short story, ‘The Seraph and the Zambesi’, that launched her fiction writing career in 1951. Bidding farewell to her investment in the Romantic imagination, Spark calls up an idea of poetic voice that draws directly on the Jewish tradition of *Ruah*. The story begins with a familiar effect of *Stimmung* as correspondence between weather, atmosphere and mood. But this Romantic motif is associated with a stifling, torpid and enervating heat whose humid effects muffle noise, like sound waves passing through water. Temporality itself appears reversed:

On the third night before Christmas I sat on the step outside my room, looking through the broken mosquito-wire network at the lightning in the distance. When an atmosphere maintains an excessive temperature for a long spell something seems to happen to the natural noises of life. Sound fails to carry in its usual quantity, but comes as if bound and gagged. That night the Christmas beetles, which fall on their backs on every step with a high tic-tac, seemed to be shock-absorbed. I saw one fall and the little bump reached my ears a fraction behind time. The noises of minor wild beasts from the bush were all hushed up, too … . Sometimes, for a moment, a shriek or cackle would hang torpidly in space, but these were unreal sounds, as if projected from a distant country, as if they were pocket-torches seen through a London fog.^97^

Cramer – a figure borrowed from Baudelaire’s tale ‘La Fanfarlo’ of 1857 and already featured in Spark’s 1952 poem ‘The Ballad of the Fanfarlo’ – is now 150 years old. As he prepares to perform the role of Seraph in a Nativity play, a real Seraph appears, a living body, but with a completed look, who orders Cramer to shut up his noise. A *contretemps* ensues, the building burns down, the Seraph disappears. The story ends as the narrator and friends walk away:

The thunder of the Falls reached us about two miles before we reached them … We came to the cliff’s edge, where opposite us and from the same level the full weight of the river came blasting into the gorge between. There was no sign of the Seraph … Then I noticed that along the whole mile of the waterfall’s crest the spray was rising higher than usual … we watched him ride the Zambesi away from us, among the rocks that look like crocodiles and the crocodiles that look like rocks.^98^

Though baffling, the story is surely an allegorical assertion of the need for a renewal and modification of the Romantic concept of imagination through new reflection on voice. The image of the thunder roaring, heard now in advance not lagging behind, the wind foaming the waters and hovering on the face of the waves, invokes Psalm 33 in the Hebrew bible where ‘God creates with the breath of his mouth’; after the apocalyptic fire, Yahweh’s breath – *Ruah* – brings energising life into the enervated scene, animating and vocalising itself through those that stop to listen.^99^The story ends with a reference to the deceptiveness of vision: that rocks look like crocodiles keeps us vigilant, but that crocodiles look like rocks may seduce us unawares.

This conception of voice is fundamental to Spark’s longest and most complex novel, *The Mandelbaum Gate*, where Barbara Vaughan remembers most vividly the ‘deep patriarchal boom’ of her Jewish grandfather.^100^ Without attempting here – for reasons of space – to offer a full reading of the novel, it is the moment when she seems most fully, as suggested earlier, to recognise the ethical implications of her conception of the auditory imagination. A crucial early event is her visit to Beersheba, the place where, in Genesis, the blind Isaac mistakenly gave his blessing to his son Jacob, disguised as Esau, his brother. But what Barbara remembers are Isaac’s words: ‘The voice is the voice of Jacob’.^101^ Isaac is deceived because once he has misheard the voice, the embodied expression of the singularity of the person, the evidence of the other senses is overwhelmed. Meanwhile, Freddy Hamilton, the British diplomat, passes through the Mandelbaum Gate, struggling and failing to keep out of his head the Hebrew chant of children’s voices heard from an upper storey: his method of repression, of ‘pitting culture against culture’, is to assert the ‘metrical precepts of Samuel Taylor Coleridge’ and allow them to chant ‘lovingly round his brain’.^102^ Barbara Vaughan too reflects: ‘I go on, she thought, with questions and answers in the old Hebraic mode, chanting away to myself.’^103^ She tries to reconcile her identities, reflecting that her Catholic faith is simply a ‘new order of an old form’, Old and New Testaments ‘bound by love into one volume’,^104^ just as the chanting of Abdul’s freedom party – Muslim, Jew and Christian – communicates in ancient rhythms that defy the nonsensical semantics of the actual words uttered. But the ethical heart of the novel lies with her experience of Eichmann, his inability to hear the voice of the other in his head, his not even ‘answering for himself’ but mimicking a ‘deity named Bureau IV-B-4 of whom he was the High Priest’.^105^ An ideal harmony of the many in one – achieved though the assertion of a shared and ancient rhythm – may still entail a failure in discernment, in listening, for it can function as a consolatory but ultimately communal solipsism that drowns out what it chooses not to hear. To hear and listen to the other, as to imagine the other hearing and listening to oneself, must remain unendingly dialogic, not preemptively resolved into harmony. Barbara need not resolve into a harmony or unity her many voices: she learns to keep open the precariously stable disequilibrium that she is and even to rejoice in that plurality. Five years later Spark would announce in her crucial essay on the desegregation of art a rejection of the empathetic imagination and an embrace of the satiric, of an art of ridicule: empathy is the art of harmonisation, but satire requires the more antagonistic art of difference that refuses identification as that merging of voices whose slippery slope might lead to the eradication of the voice of the other altogether. The capacity to listen and discern is a difficult one, and for Spark at least, even then, not in itself a guarantor of ethical action: but the novelist’s responsibility must surely be to make us listen more carefully, to alienate us from a comfortable alienation.

## Living alone together: voices, singularity and the practices of *colligere*

Elias Canetti’s *Crowds and Power* (1961) finishes with an account of the psychotic German judge, Daniel Paul Schreber, and the teeming voices chattering inside his head relieved only by the loud bellowing that seemed to give him temporary respite. Canetti’s analysis of Schreber bears close similarities to George Herbert Mead’s earlier recognition of the processes of internalisation and reflexivity that allow the voices of the crowd, as the ‘generalised other’, dialogically to enter and inflect the flow of inner speech that normally brings self – inherently therefore an interpellated self – to presence to itself. In recognising the crowd that is enfolded within the individual and then interleaved without, Mead and Canetti posit a self that is inherently reflexive, a singularity and a crowd, a me and not-me; an I that is a we.^106^ Spark’s post-modern reflexivity, however, is honed by the group rather than the crowd. So she mostly builds auratic architectures of spaces more contained than the streetwalks and soundscapes of the modernist city: the rooming house, hospital ward, office, school and factory are her sounding boards. Like her contemporary, Auden, who advocates a new ‘civitas of sound’ in a noisy megaphone culture of political rhetoric and loudspeakers, Spark’s exploration of groupthink is an education in learning to listen and to hear the voices of the aged, the unmarried, the genteelly impoverished, the stateless, the white-collar worker. Auden’s sense of a world unattuned to quietness, that has lost the ability to listen because ‘each ear/ Is listening to its hearing, so none hear’, is also Spark’s.^107^

Two of her most accomplished novels, *The Girls of Slender Means* and *A Far Cry from Kensington*, are located in rooming houses whose architectures are built almost entirely through juxtapositions and counterpoints of sounds and voices. In *The Girls of Slender Means*, set in 1945, class and age are the chief ordering principles of the household; in *A Far Cry* (set in the early to mid-50s), what appears to be a more cosmopolitan and democratic if pandemonic background hum – born of the new post-war hybrid vocality of the dislocated, the refusenik and the class refugee – is punctuated by the screams of the radically and genuinely dispossessed, the terrorised and the entirely stateless. Both novels are presented in the form of recollection or, more accurately, re-collection. Their narrators listen in to and bring the past into presence, dissolve and resurrect the boundaries between the mind’s ear in its activity of recall and the presence of the past as it is reshaped through auditory imagination into a new and meaningful soundscape.

*A Far Cry* begins with its narrator Nancy (once the Mrs Hawkins of the story), lying in bed, listening in and recollecting the past, now thirty years far away from the turmoil of office life – ‘my job was the noisiest I’ve ever known’ – and the cacophony of her old lodging house, with its clatter, chatter, telephones, radio, televisions, hoovers, rows, banging, of different lifestyles.^108^ Layered with earlier acts of memorisation, she recalls lying awake at night, ‘listening to the silence … I heard the silence’.^109^ As her mind wanders in the dark, conflating then and now, she asks,

Can you decide to think? Yes, you can. You can put your mind to anything most of the time. You can sit peacefully in front of a blank television set, just watching nothing; and sooner or later you can make your own programme much better than the mass product.^110^

Mrs Hawkins is as serious in her task as Proust’s Marcel, highlighting memory as composition, ordering, layering, harmonising, but with an ethical orientation:

listening to the silence, prefiguring the future, picking out of the past the scraps I had overlooked, those rejected events which now came to the foreground, large and important, so that the weight of destiny no longer bore on the current problems of my life.^111^

Consciously and attentively listening to and reordering the past to bring out its hidden significance, she builds a house of fiction, now discerning the emergence of patterns that carry a moral weight. As in *The Comforters*, there is a metafictional doubling, but here Spark intuits an analogy between the novelist’s art and the specifically pre-modern, indeed medieval, Kataphatic practice of creative thinking as the modelling of memory palaces. Through careful sifting and reordering a mental inscape is built: ‘listening to the silence with my outward ears and to a crowding-in of voices with my inward ear’.^112^ Mary Carruthers, names this a ‘craft of thought’, a means of coping with and coming to understand the suffering of others, one’s own mortality, but a process too of renewal of the life of the spirit.^113^ This practice, of *inventio* – bringing together invention, inventory and intention or *intentio* – involved the placing of memories in locations whose analogical associations provide points of metaphorical transfer for ethical meditation and reflection, ‘where *memoria* and invention come together in a single cognitive process’, a repudiation of the merely imitative or mechanically mimetic.^114^ First comes a process of *collocare*, gathering up, next the business of *colligere*, building a pattern: the material is intimately entwined with the moral.^115^ So in *Memento Mori*, the geriatric ward provides an opportunity to meditate on mortality and dying, just as the Marcia Blaine School provides the opportunity for reflection on paideia, or the ethical practice of education. Even in its title, *The Girls of Slender Means* plays directly on the relation between the material and the moral. Spirit is not much in evidence: inspiration is reduced to Jane Wright’s ‘brain-waves’, the ecstatic to Nicholas Farrington’s account of Freudian sublimation.^116^ Slenderness reflects the ethos of a patriarchal society where attracting a man is the telos of femininity, just as the bartering of ration coupons reflects a political economy where any remnant of civitas, of the ‘good’, is absorbed into the imperatives of rational economics and Darwinian competition for scarce resource.

This meditative tradition is reworked in the spiritual exercises of Ignatius, where in the composition of place too the mind is tuned up or heightened to experience the presence of God. *Tonos* indicated both tuning in and tuning up, a process of tightening the strings, heightening the body and its affects towards a state of concentration to raise the inscape to a higher plane for moral and allegorical meditation. In this novel, *tonos* is provided in Joanna’s rhapsodic proclamations of poetry, mimetic performances that are seen to give ‘culture’ and raise the ‘tone’ in order to impress visiting males. Similarly, Selina’s mantra of perfect poise, balance and tone, is an *aide-memoire* to the bodily self-discipline needed to acquire a man and find one’s way to the good life of monied elegance.^117^ Even the other-worldliness of Joanna’s passionate vocalisations of poetry is compromised: lessons in elocution – correct vowels and intonation patterns – purchased by aspirational young women as key to upward mobility. No one, least of all Nicholas Farrington – or at least not yet, until after her death when her voice haunts most of the characters with its final words of the Psalms and proclamation of Hopkins ‘Wreck of the Deutschland’ with its tall and sacrificial nun – pays close and careful attention to her words. Before her death as he hears her voice competing with the radio and imagines her proclaiming, she is frozen into a figure on a Grecian Urn – another still unravished bride of quietness. Soon after, now proclaiming the evening psalter for Day 27 in her trance-like state of absorption, Joanna fails to hear the megaphone warning and falls with the burning house, surely suicidally, to her death. Like Plato’s Ion, her repertoire is limited. Spark offers another model of the imagination that will no longer suffice: everyone hears but no one listens until it is too late because, like her Greek counterpart, this rhapsode too fails to provide or elicit any critical context for reinterpretation or relevance until after her death. Unlike Spark’s novel, with its recomposition of the past and its complex and emergent pattern of vocal juxtapositions, Joanna’s is not an art for mneme, but a mimetic performance.

Just as Sandy Stranger notes the compositional economy of Teddy Lloyd’s paintings – that each portrait of the Brodie set looks also like Miss Brodie – so the principle of economy in monastic thoughtcraft – one of layering, juxtaposing, building, creating composites so that patterns emerge through the process of attentive and disciplined inner sensing – evidently informs the art of Spark’s poetic composition. And it is what is singularly missing in the activity of her many copiers, reporters, transmitters, informers, informants, impersonators, ventriloquists. For her art too is oriented to a civic responsibility to provide patterns for communal thinking, engaging affect, form, the discipline of inward attention, in order to raise and goad feeling: to pierce, scar, puncture: the idea of ‘*punctus*’ as wounding the page with one’s pen.^118^ Such notions, associated with mystical thinkers, urge a renunciation of the art of empathy for one of satire and ridicule that can leave a ‘salutary scar. It is unnerving. It can paralyse its object’.^119^ Spark’s compositors never recollect in tranquility: restless, disturbed, they are haunted by voices and sounds never previously heard at all. The scream, piercing, a *punctus*, is the voice of the outcast, the scapegoat, those shut out of the house but who refuse to be resolved into the convenient harmony and habitus of the one, the group : Sandy Stranger, gripping the bars of her grille but haunted too by the ‘whine’ of Miss Brodie’s voice; Lise screaming in four languages as her murderer plunges in the knife; Mary Macgregor screaming hither and thither as the fire engulfs her.

Reflecting on Muriel’s evident enjoyment of her time at Milton Bryan, working for the PWE, part of a loose community and yet, essentially, solitary, Martin Stannard notes how ‘what she liked best was the supporting structure of a communal life which left her alone to think and to write. She did not crave intimacy’.^120^ In *How to Live Together*, a posthumously published work based on his late lectures, Roland Barthes, in search too of a place where one might preserve singularity, creative solitude, but within a rich community – living alone together – revisited the lifestyle of the fourth century monks at Mount Athos: what they discovered, he claims, was an ‘idiorrhythm’, preserving singularity but contributing to the rhythmic harmony of the group.^121^ That structure was discovered between the withdrawn solitariness of the eremitic visionaries, the desert fathers, and the later strict coenobitism of the Benedictine Rule. Coenobium for Barthes is assimilation without freedom. Like Spark, he searches for models of the group that might allow the idiorrhythmic to flourish in the modern world so that solitude might become solicitude, or care for a community beyond the self, but always listening to the difference that opens up a space for critical judgement and not simply identification. Spark’s ethics are far from those of a liberal pluralism that slips easily into the careless ethical relativism of a postmodern anything goes. But neither is she caught up in the crowd in thrall to the authoritative or acousmatic voices of those whose harmonies facilitate a necropolitics. Barthes’ text analyses Bion’s 1961 *Experiments in Groups*, where the psychoanalyst experimented with the possibility of emergence of a group mind not organised by rule but governed only by the heterorhythmic as the subversion of centralised authority and power. Even as she satirises New Age political thinking and the cacophonous anarchisms of the 1950s and 1960s, Spark uses her novels to explore ways of conceiving community based on attunement, listening, the possibilities of the idiorhythmic but also the capacity to hear dissonance, dissent, the voice that has been masked by the habitual roar and mumble. Having reviewed more or less positively Eliot’s *Notes Towards the Definition of Culture* (1948), his riposte to the rationalism of the post-war planners with their calculative means-end thinking, Spark makes a further response in *The Girls of Slender Means*.^122^ Nicholas Farrington’s half-hearted anarchism that knowingly imposes on the May of Teck an image sentimental and deeply unfamiliar to itself, is satirised in the novel as the careless and contradictory listening of the Fitzrovian poet manqué, incidentally working for British Intelligence and writing an anarchist treatise defending the monarchy, whilst striking a radical pose to elicit sexual favours from beautiful women. His is another example of the undisciplined imagination. But for Spark, Eliot’s idea of circumventing the new culture of planning by reviving a feudal culture of *noblesse oblige* underpinned by a faith in Christian redemption, won’t do either. In Eliot’s image of the auditory imagination, the new and the ancient might be fused through the feeling for syllable and rhythm. But for Spark that process must not be segregated, by class, gender or genre; art must connect with the world outside in the hope of making something better. In refashioning the auditory imagination as a place where the voices of the other might be listened to and heard, Spark recognises that the ideal community, as a place of living alone together must begin with the capacity of the imagination to create complex inner presences, to turn concepts into percepts, and to hear the ragged or jangling or whispered voice of the other in its own. In this revocalised dialogicality of thought and of thoughtfulness, one might at least challenge the monologic and the monotonous, the ontologically narrow and the bureaucratically closed, and understand that without this mode of mindfulness, the voice of the other cannot be listened to, for it would never have been heard at all.

## Notes

1. T. S. Eliot, *The Uses of Poetry and the Uses of Criticism* (Cambridge, MA: Harvard University Press, 1933), p. 111.

2. Muriel Spark, *Curriculum Vitae* (London: Constable, 1992), pp. 203, 204. As early as February 1944 though, she notes that, for the first time ‘in ages’ she buys poetry books in Cape Town and reads T.S Eliot’s *The Dry Salvages*, ‘on that dangerous journey’ (p. 140).

3. *Curriculum Vitae*, p. 206.

4. The episode is revisited briefly in *A Far Cry*, but conveyed with much more foregrounding of disturbing experiences of hearing voices. Mrs Hawkins’ breakdown and her inability to deal with the noisiness of the world is described as ‘the choking hysteria in the London voices around me’, like the voices that later ‘crowd into her mind’ in solitude, p. 112.

5. Marguerite Sèchehaye, *Autobiography of a Schizophrenic Girl* (New York: Signet, 1970), p. 9.

6. Spark, *Curriculum*, p. 206.

7. Ibid.

8. Ibid.

9. Sara Frankel, ‘An Interview with Muriel Spark’, *Partisan**Review*, 54 (Summer 1987), p. 450.

10. It might be argued that this was a similar moment in the history of the novel, not simply with the rise of The Movement and the social realism of the various Angry Young Men, but the beginning of the campaign by C.P. Snow, Pamela Hansford Johnson, and others, for a return to realism in fiction.

11. Victor Tausk, ‘On the Origin of the “Influencing Machine” in Schizophrenia’, *Psychoanalytic Quarterly*, 2 (1933), pp. 529–30.

12. Spark, *The Comforters* (London: Virago, 2009).

13. Muriel Spark, ‘My Conversion’, *Twentieth Century*, 170 (1961), p. 60.

14. Edmund Wilson, *The Wound and the Bow* (Oxford: Oxford University Press, 1941).

15. Spark, *Curriculum*, p. 115.

16. Spark, ‘My Conversion’, p. 61

17. ‘The Catholic belief is a norm from which one can depart’, Spark, ‘My Conversion’, p. 60; for her ‘nevertheless principle’, see ‘What Images Return’, *The Informed Air*, pp. 11–7.

18. Muriel Spark, ‘The Desegregation of Art’, in *The Informed Air: Essays* (New York: New Directions, 2014), pp. 77–82.

19. Muriel Spark, *The Mandelbaum Gate* (Harmondsworth: Penguin, 1967), p. 177.

20. Spark discussed these periods of her life in *Curriculum Vitae* but also in autobiographical essays such as ‘Edinburgh Born’, *New Statesman*, 10 August 1962, 180. For the most detailed account, see Martin Stannard, *Muriel Spark: The Biography* (London: Weidenfeld and Nicholson, 2009).

21. Spark, *The Mandelbaum Gate*, p. 28.

22. Spark, *Curriculum*, pp. 27, 25.

23. Ibid., 152.

24. See ‘The Gentile Jewesses’, in *The Stories of Muriel Spark* (London: The Bodley Head, 1985), pp. 270–77; also ‘Note on My Story “The Gentile Jewesses”’, in Muriel Spark, *The Informed Air,* pp. 5–7. Spark wrote extensively on the Romantics: see for example, Muriel Spark*, Child of Light. A Reassessment of Mary Wollstonecraft Shelley* (Hadleigh: Tower Bridge Publications, 1951) and with Derek Stanford, *Tribute to Wordsworth* (London: Wingate, 1950).

25. Muriel Spark, *Loitering with Intent* (London: Triad/Granada, 1982), p. 14.

26. William Wordsworth, *The Collected Poems* (London: Wordsworth Editions, 1994), p. 633.

27. Spark, *Loitering*, p. 49.

28. I am using the term presence in its phenomenological sense: see Maurice Merleau-Ponty, *Phenomenology of Percepti*on, trans. Donald A. Landes (London: Routledge, 2012) and *The Primacy of Perception*, ed. J.M. Edie (Evanston: Northwestern Press, 1964); for an account of intentionality and subjectivity, see Dan Zahavi, *Self and Other: Exploring Subjectivity, Empathy and Shame* (Oxford: OUP, 2014); for an account of intentionality and hallucinations, see J. Parnas et al., ‘Examinations of Anomalous Self-experience’, *Psychopathology*, 38, pp. 236–58 and Matthew Ratcliffe, *Real Hallucinations: Psychiatric Illness, Intentionality, and the Interpersonal World* (Cambridge, MA and London: MIT Press, 2017), pp. 19–28.

29. Muriel Spark, *The Girls of Slender Means* (Harmondsworth: Penguin, 1966), p. 7.

30. Ibid.

31. Ibid., p. 10.

32. Ibid., p. 28.

33. Virginia Woolf, ‘A Sketch of the Past’, in Jeanne Schulkind (ed.), *Moments of Being* (New York: Harvest, 1985), p. 67.

34. Spark, *Loitering*, p. 81

35. ‘The First Year of My Life’, *The Stories of Muriel Spark*, pp. 263–70.

36. Muriel Spark, *Voices at Play: Stories and Ear-Pieces* (London: Macmillan, 1961), p. v.

37. Muriel Spark, *The Prime of Miss Jean Brodie* (London: Penguin Classics, 2000), p. 21.

38. Spark, *Child of Light*, p. xii.

39. Derek Stanford, *Muriel Spark* (London: Open Gate, 1963), p. 15.

40. Spark, *The Informed Air*, p. xi.

41. Joseph Hynes (ed.), *Critical Essays on Muriel Spark* (New York: G. K. Hall, 1992), p. 31.

42. The acousmatics were the followers of Pythagoras who were forbidden from seeing their master, only allowed to hear his voice from behind a curtain. The term was revived by Pierre Shaeffer, the founder of musique concrete in the late 1940s. His writings on the ‘acousmatic’, informed by phenomenology, are gathered in his collection, *Traité des Objets Musicaux: Essais interdisciplines* (Paris: Seuil, 1966).

43. Muriel Spark, ‘The Religion of an Agnostic: A Sacramental View of the World in the Writings of Proust’, *Church of England Newspaper*, 27 (November 1953), p. 1.

44. Marcel Proust, *In Search of Lost Time*, trans. C.K. Moncrieff and T. Kilmartin (London: Everyman, 2001), Vol. 3, p. 419.

45. Muriel Spark, *A Far Cry from Kensington* (London: Penguin, 1988), p. 120.

46. Ibid., 148.

47. Muriel Spark, *Not to Disturb* (Harmondsworth: Penguin, 1974), 96.

48. Aristotle, *Politics*, 1253a9–19.

49. *The Comforters*, p. 27.

50. Muriel Spark, *The Hothouse by the East River* (Harmondsworth: Penguin, 1973), p. 12.

51. Spark, *A Far Cry*, p. 63.

52. Ibid., p. 73.

53. Bruno Latour, *On the Modern Cult of the Factish Gods* (Durham and London: Duke University Press, 2010).

54. See Bruno Latour and Steve Woolgar, *Laboratory Life: The Construction of Scientific Facts* (Beverly Hills, CA: Sage, 1979).

55. *The Abbess of Crewe* (London: Macmillan, 1974), pp. 103–4.

56. Eugen Bleuler, *Dementia Praecox, or the Group of Schizophrenias*, trans. J. Zinkin (New York: International Universities Press, 1950), pp. 123–5.

57. Virginia Woolf, ‘An Unwritten Novel’, in Susan Dick (ed.), *The Complete Shorter Fiction* (London: Triad/Grafton Books, 1991), p. 120.

58. Spark, *The**Informed Air*, p. 39.

59. *The Comforters*, p. 53.

60. Ibid., p. 45.

61. Muriel Spark, *John Masefield* (London: Peter Nevill, 1953), p. 68.

62. See Eugen Fischer, *Philosophical Delusion and Its Therapy* (London: Routledge, 2011); and Fiona Macpherson and Dimitris Platchias, *Hallucination: Philosophy and Psychology* (Cambridge, MA and London: MIT Press, 2013).

63. For a discussion of debates around the ‘ocularcentric’, see Martin Jay, *Downcast Eyes: the Denigration of Vision in Twentieth-Century French Thought* (Berkeley: University of California Press, 1993).

64. The first extended phenomenological account of sound is Don Idhe, *Listening and Voice: Phenomenologies of Sound* (New York: State University of New York Press, 2007).

65. Spark, *Loitering*, p. 61.

66. Mrs Hawkins’ term for Hector Bartlett in *A Far Cry*, p. 45.

67. See especially, Vassiliki Kolocotroni, ‘Poetic Perception in the Work of Muriel Spark’, in Michael Gardiner and Willy Maley (eds), *The Edinburgh Companion to Muriel Spark* (Edinburgh: Edinburgh University Press, 2010), pp. 16–26.

68. Frank Kermode, ‘Old Testament Capers’, *LRB*, 6, no. 17 (1984), p. 11.

69. See, in particular, Walter J. Ong’s *The Presence of the Word* (New Haven, CT and London: Yale University Press, 2009).

70. For a discussion of the development of the vodocoder, see Friedrich A. Kittler, *Gramophone, Film, Typewriter* (Stanford: Stanford University Press, 1999).

71. Sefton Delmer, *Black Boomerang: An Autobiography* (Secker and Warburg, 1962), Vol. 2, p. 103.

72. Spark, *A Far Cry*, p. 141.

73. Roland Barthes, ‘The Death of the Author’, in Stephen Heath (ed.), *Image, Music, Text* (London: Fontana, 1977), p. 142.

74. Plato, *Republic*, 607a.

75. See Adriana Cavarero’s discussion, ‘The Rhapsodic Voice; or Ion’s Specialty’, in *Towards a Philosophy of Vocal Expression*, trans. Paul A. Kottman (Stanford: Stanford University Press, 2005), pp. 79–95.

76. *The Comforters*, p. 186.

77. Ibid., p. 11.

78. Ibid., pp. 15, 16.

79. Ibid., p. 4.

80. Ibid., p. 168.

81. Ibid., p. 166.

82. Ibid., p. 53.

83. *The Comforters*, p. 85.

84. Ibid., p. 4.

85. Ibid., p. 52.

86. Ibid., p. 22.

87. Ibid., p. 92.

88. Ibid., p. 126.

89. Ibid., p. 167.

90. Ibid., p. 37.

91. Ibid., p. 89.

92. See earlier, Spark, ‘My Conversion’.

93. ‘The Cloud of Unknowing’, a fourteenth century mystical text of unknown authorship, is a key anaphatic text of Christian mysticism, asserting the limitation of reason in knowing God, and the importance of kenosis as a preparation for ‘knowing’ God through love. For an account of the importance of the Kataphatic as inner presencing in contemporary American evangelism, see Tanya Luhrmann’s fascinating and erudite study, *When God Talks Back* (New York: Knopf, 2012).

94. *The Spiritual Exercises of St Ignatius of Loyola*, trans. Father Elder Mullan (New York: P.J. Kennedy, 1914).

95. Spark, *The Mandelbaum Gate*, p. 26.

96. See, for example, Benjamin’s, ‘God breathes his breath into man: this is at once life and mind and language’ and not the ‘bourgeois’ idea of language as primarily of semantic communication: in *Selected Writings*, ed. M. Bullock and Michael W. Jennings (Cambridge: Harvard University Press, 1996), Vol. 1, p. 67.

97. Muriel Spark, ‘The Seraph and the Zambesi’, in *Stories*, p. 78.

98. Ibid., pp. 83–4.

99. In her memoir, Spark describes her first experience of the Victoria Falls on the Zambesi River as a ‘mystical experience’, hearing the ‘tumult’ and the ‘roar’ of the waters and invoking the local description of it – Musioa-tunya-‘the smoke that thunders’. She reflects too how they became hear a ‘symbol of spiritual strength’: Spark, *Curriculum*, pp. 128–9.

100. Spark, *Mandelbaum Gate*, p. 35.

101. Ibid., p. 24.

102. Ibid., p. 15.

103. Ibid., p. 50.

104. Ibid., p. 38.

105. Ibid., p. 179.

106. George Herbert Mead, *Mind, Self and Society* (Chicago, IL: Chicago University Press, 1933).

107. W.H. Auden, ‘At the Party’, first published in 1961, *Collected Poems* (New York: Vintage, 1991), p. 737.

108. Spark, *A Far Cry*, p. 12.

109. Ibid., p. 5.

110. Ibid.

111. Ibid.

112. Ibid., p. 112.

113. Mary Carruthers, *The Craft of Thought: Meditation, Rhetoric, and the Making of Images*, *400–1200* (Cambridge: Cambridge University Press, 1998).

114. Carruthers, *The Craft of Thought*, p. 16.

115. See Mary Carruthers, ‘The Poet as Master Builder: Composition and Locational Memory in the Middle Ages’, *New Literary**History*, 24 (1993), pp. 881–904.

116. Spark, *Girls of Slender Means*, p. 45.

117. Ibid., p. 50.

118. See Mary Carruthers, *The Book of Memory* (Cambridge: Cambridge University Press, 1990).

119. Spark, *The Informed Air*, p. 81.

120. Stannard, *Muriel Spark*, p. 71.

121. Roland Barthes, *How to Live Together*, trans. Kate Briggs (New York: Columbia University Press, 2002), p. 7.

122. Muriel Spark, ‘The Poet in Mr. Eliot’s Ideal State’, *Outposts*, 14 (Summer 1949), 26–8.

